# Complex G-protein signaling of the adhesion GPCR, ADGRA3

**DOI:** 10.1016/j.jbc.2025.108441

**Published:** 2025-03-22

**Authors:** Sofie M. Bagger, Hannes Schihada, Anna L.S. Walser, Anna K. Drzazga, Lukas Grätz, Tiago Palmisano, Christina K. Kuhn, Maša Mavri, Ann-Sophie Mølleskov-Jensen, Gregory G. Tall, Torsten Schöneberg, Signe J. Mathiasen, Jonathan A. Javitch, Gunnar Schulte, Katja Spiess, Mette M. Rosenkilde

**Affiliations:** 1 Department of Biomedical Sciences, Faculty of Health and Medical Sciences, University of Copenhagen, Copenhagen, Denmark; 2 Department of Physiology and Pharmacology, Section of Receptor Biology and Signaling, Karolinska Institutet, Stockholm, Sweden; 3 Departments of Psychiatry and Molecular Pharmacology and Therapeutics, Columbia University Vagelos College of Physicians and Surgeons, New York, New York, USA; 4 Division of Molecular Therapeutics, New York State Psychiatric Institute, New York, New York, USA; 5 Molecular Biochemistry, Medical Faculty, Rudolf Schönheimer Institute of Biochemistry, Leipzig, Germany; 6 Department of Pharmacology, University of Michigan School of Medicine, Ann Arbor, Michigan, USA

**Keywords:** ADGRA3, cAMP response element-binding protein (CREB), class B2 adhesion GPCR (aGPCR), G protein–coupled receptor (GPCR), G protein–mediated cell signaling, GPR125

## Abstract

ADGRA3 (GPR125) is an orphan adhesion G protein–coupled receptor (aGPCR) involved in planar cell polarity, primarily through recruitment of the signaling components disheveled (DVL) during vertebrate gastrulation and discs large homolog 1, implicated in cancer. Limited knowledge exists of the canonical G protein–coupled receptor pathways downstream of ADGRA3. Here, we employed a series of human cell line–based signaling assays to gain insight into the G protein–mediated signaling of ADGRA3. We designed ADGRA3 constructs based on transcript variant analysis in publicly available human liver and brain RNA-seq datasets. Cleavage in the GPCR autoproteolysis site (GPS) is an aGPCR hallmark; thus, we generated a truncated ADGRA3 (C-terminal fragment, CTF) corresponding to a potential cleavage at the GPS. We found low-level activation of Gi and Gs by ADGRA3 and slightly more by its CTF. As the N terminus of the CTF constitutes a class-defined tethered agonist (so-called stachel peptide), we removed the initial three amino acids of the CTF. This resulted in abrogated G protein–mediated signaling, as observed for other aGPCRs. Due to the central role of ADGRA3 in planar cell polarity signaling through DVL recruitment, we investigated the G-protein signaling in the absence of DVL1-3 and found it sustained. No transcriptional activation was observed in an assay of downstream β-catenin activity. Collectively, this establishes classical G protein–mediated signaling for ADGRA3.

Adhesion G protein–coupled receptors (aGPCRs) constitute class B2 of the superfamily of G protein–coupled receptors (GPCRs). As a distinct class of receptors, aGPCRs regulate processes in various physiological systems, including the nervous and musculoskeletal systems, and play curtail roles in metabolism, immunity, and development (1). Mutations and dysregulation of aGPCRs in humans are associated with diverse diseases, including developmental defects, neurological disorders, and cancer (1). Signal transduction from aGPCRs spans coupling to heterotrimeric G proteins and diverse alternative pathways, including β-arrestin recruitment and β-catenin–mediated signaling engagement (2, 3). aGPCRs are divided into nine families based on their phylogenetic relations, of which family III comprises ADGRA3 (previously known as GPR125) in addition to ADGRA2 (GPR124) and ADGRA1 (GPR123) (4, 5). ADGRA1-3 are all expressed in the brain and linked to developmental signaling pathways (6, 7, 8, 9, 10, 11, 12). Furthermore, ADGRA3 expression is associated with various cancers showing both oncogenic and tumor-suppressive effects (13, 14, 15, 16).

ADGRA3 was initially identified as a spermatogonial stem cell marker (17), and the knockdown of ADGRA3 in male mice leads to obstructive azoospermia with high penetrance (18), while female mice lacking ADGRA3 develop a closed vagina to a similar extent (12). In mammary progenitors, ADGRA3 expression congregates at ductal tips during morphogenesis, and high expression predicts early tumor onset and poor outcome (13). In lacrimal gland progenitors, its expression segregates to the tip of migrating embryonic lacrimal ducts, and its absence leads to dry eyes and blepharitis (19). Further, ADGRA3 plays a role in adipose thermogenesis as overexpression or activation-induced expression stimulates browning of adipocytes (20). At the cellular level, ADGRA3 contributes to planar cell polarity (PCP) regulation of the zebrafish embryo through redistribution of the scaffolding phosphoprotein disheveled (DVL) (11, 21). ADGRA3 also interacts with the scaffolding protein discs large homolog 1 (Dlg1) through its C-terminal ETTV domain (Glu^1318^-Thr^1319^-Thr^1320^-Val^1321^) (21, 22, 23). Moreover, the knockdown of ADGRA3 in osteoclasts downregulates receptor activator of nuclear factor kappa-B ligand–induced pathways, which are important for osteoclastogenesis (24).

aGPCRs contain large extracellular N termini harboring several adhesive domains and the conserved GPCR autoproteolysis-inducing domain comprising a GPCR proteolytic site (GPS) motif. The GPS in most ADGRA3 vertebrate orthologs is SL|S/G (where | denotes the potential cleavage point). Although this differs from the canonical GPS motif (HL|S/T), autoproteolytic cleavage of ADGRA3 was suggested based on immunoblot fragment sizes, yet the precise location of the cleavage site was not ascertained (21). In general, upon aGPCR autoproteolysis, the fragments remain noncovalently associated as a heterodimer: an extracellular N-terminal fragment (NTF) and a C-terminal fragment (CTF) with the heptahelical transmembrane GPCR fold (Fig. 1*A*) (25, 26). As a general concept, removing the NTF at the GPS results in enhanced CTF signaling relative to the full-length (FL) receptor, suggesting a suppressive activity of the NTF among aGPCRs (27). Furthermore, evidence is building towards a role of the class-defined so-called *stachel* peptide immediately downstream of the GPS to work as an intramolecular tethered agonist on aGPCRs, internally controlling receptor activity (28, 29, 30, 31). Moreover, alternative splicing events and multiple transcript variants are reported for several aGPCR class members, some with tissue-specific expression, implying that caution should be taken when designing and evaluating ectopic (over)expression studies (32, 33).

**Figure 1:**
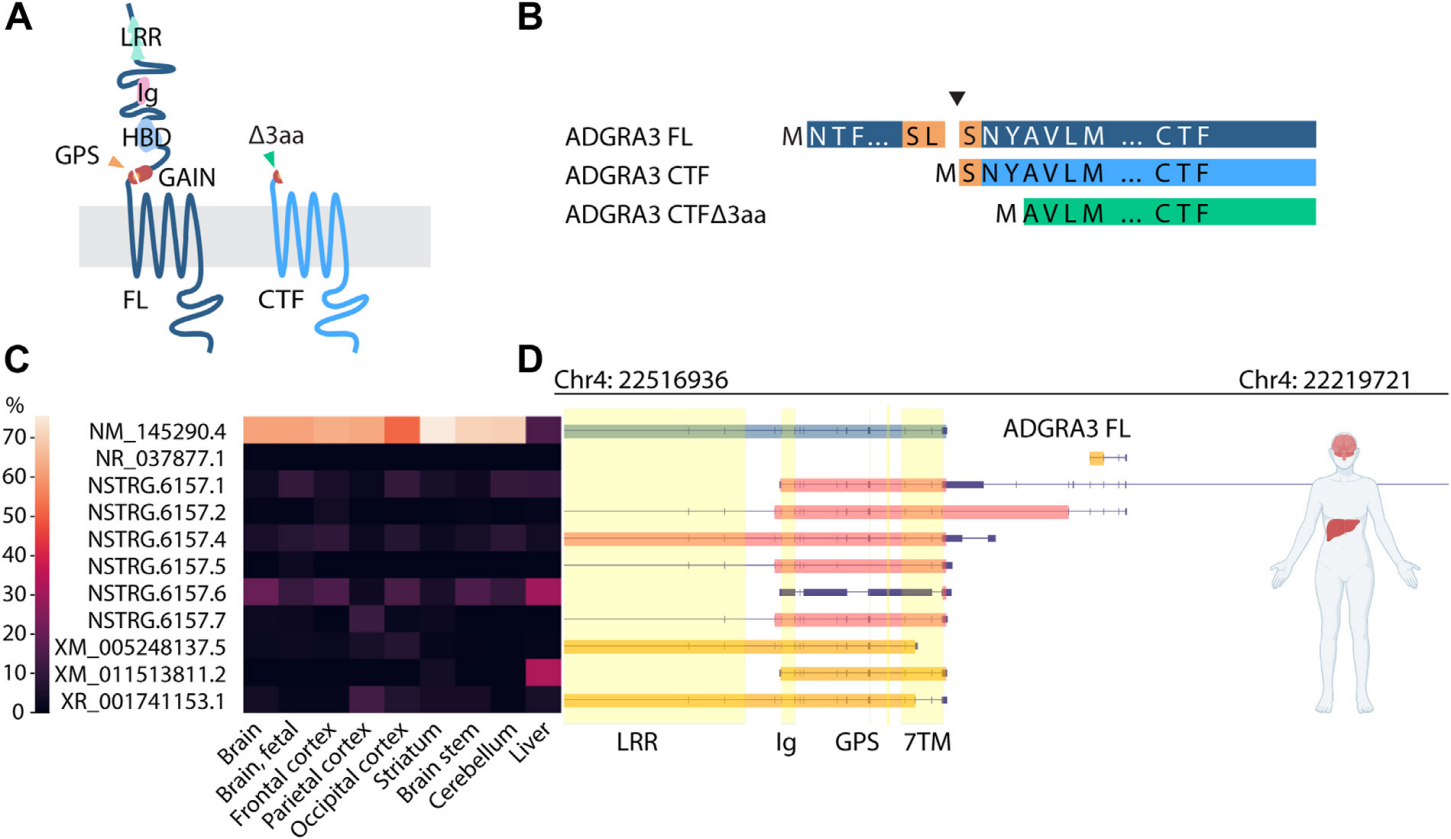
**ADGRA3 transcript variant analysis and construct design.***A*, schematic outlining of the tertiary structure of the ADGRA3 full-length (FL) construct, based on the NM_145290.4 transcript variant and the corresponding C-terminal fragment (CTF) construct, truncated at the GPS. *Green triangle* symbolize the site of Δ3aa truncation of CTF. *B*, outline of the sequences of ADGRA3 constructs used throughout the study. The *triangle* depicts the site of CTF truncation between Ser^736^-Leu^737^ and Ser^738^. *C*, RNA-seq data from human brain and liver were analyzed for transcript variant disposition reference-guided transcript assembly with concepts of *de novo* by the pipeline described in Experimental procedures. The heat map shows the abundance of each transcript variant as a percentage of all variants according to the scale. NM_145290.4 is predominant. Novel accession numbers starting with “NSTRG” are not yet annotated in the NCBI database. *D*, visualization of the transcript variants at locus Chr 4: 22219721-22516936, showing the longest ORFs in *thick boxes* for novel (*red*) and database annotated (*orange*) transcripts. The predominant transcript variant NM_145290.4, containing 19 exons, is highlighted in *blue*. *Thinner purple boxes* depict 3′- and 5′ UTRs, and *dark blue lines* are introns. *Yellow columns* show regions encoding ADGRA3 protein domains and the GPCR proteolytic site (GPS). Aa, amino acid; Chr, chromosome; GAIN, GPCR autoproteolysis-inducing domain; HBD, hormone-binding domain; Ig, immunoglobulin-like domain; LRR, leucine-rich repeat domain; 7TM, seven helix transmembrane domain; TPM, transcripts per million.

Here, we probe the downstream signaling of ADGRA3 through heterotrimeric G proteins. First, we analyzed the transcript variants of ADGRA3 in publicly available human liver and brain RNA-seq datasets, which guided the design of ADGRA3 constructs employed in this study. Next, we generated a truncated ADGRA3 construct where the NTF was removed, corresponding to the functional cleavage of the receptor at the GPS site, to investigate the ability of ADGRA3 and its potential CTF to initiate signaling through heterotrimeric G proteins (Fig. 1, *A* and *B*). ADGRA3 interacts physically with Gα_i_, but so far, this has not been verified functionally (21). Therefore, we systematically studied ADGRA3-mediated G-protein activation. Due to the previously established interaction with DVLs (11, 21), we used cells with and without DVLs (ΔDVL1-3) (34) to probe G-protein signaling dependency on the presence of DVL. Lastly, we investigated the effect of truncating the putative intramolecular “tethered” agonist within the ADGRA3 CTF. Taken together, our data shed light on G protein–mediated signaling by ADGRA3, which is hitherto unreported for class III aGPCRs.

## Results

### One dominant transcript variant of ADGRA3 is expressed in the human liver and brain

The genomic architecture of aGPCRs is often complex, each coding sequence stretching over numerous exons, allowing alternative splicing events that produce multiple transcript variants (32, 35). Since the various transcript variants from an aGPCR gene can exhibit significantly different functions and tissue expression profiles (36, 37, 38), we sought the most prominent ADGRA3 transcript variant for further analysis. Therefore, we performed reference-guided transcript assembly in publicly available human RNA-seq datasets from eight regions of the brain and the liver of deeply sequenced (7–111 million reads per sample) RNA-seq data (39, 40, 41). The pipeline used for this assembly is delineated in the Experimental procedures section, with Gene Expression Omnibus accession numbers available in Table S1. We found eleven transcript variants that met the cutoff for being expressed significantly in at least one of the tissues (Fig. 1*C*). Of those, five variants are already annotated in the NCBI database (ORFs marked orange), and six were novel (ORFs marked red). One transcript variant (annotated NM_145290.4, ORF marked blue) presented with the highest expression over all analyzed brain regions, accounting for 51.6% to 75.5% (in brain occipital cortex and striatum, respectively) of the expressed variants, and showed high absolute expression levels (Table S2). This variant contains 19 exons and encompasses all the annotated domains of ADGRA3 (Fig. 1, *A* and *D*). In the liver, NM_145290.4 accounted for 15.1%, whereas the two major liver variants were NSTRG6157.6 and XM_011513811.2, accounting for 30.6% and 32.8% of total variant expression, respectively. However, upon examining the sequences, we found that NSTRG6157.6 is spurious, with only a very short ORF and no signal peptide. We predicted 1 hydrophobic stretch of signal peptide character in XM_011513811.2, yet short and of unknown targeting potential (Fig. 1*D*). Collectively, this highlighted NM_145290.4 as the most relevant and highest expressed ADGRA3 transcript variant in the analyzed human tissues.

Based on the dominance of the 19-exon transcript variant across the range of analyzed human tissues, we continued with NM_145290.4 as the template for our construct design for transient transfection of human cell lines, here referred to as ADGRA3 FL (Fig. 1, *A* and *B*). We truncated ADGRA3 FL at the predicted GPS cleavage site (marked in Fig. 1, *A* and *B* by orange color and a gap) to generate an ADGRA3 CTF construct for the signaling analysis (21). Further truncations were introduced to abolish the putative tethered agonist (ADGRA3 CTFΔ3aa) (Fig. 1, *A* and *B*, marked by green color).

### Broad screen for G-protein activity downstream of ADGRA3

**Figure 2:**
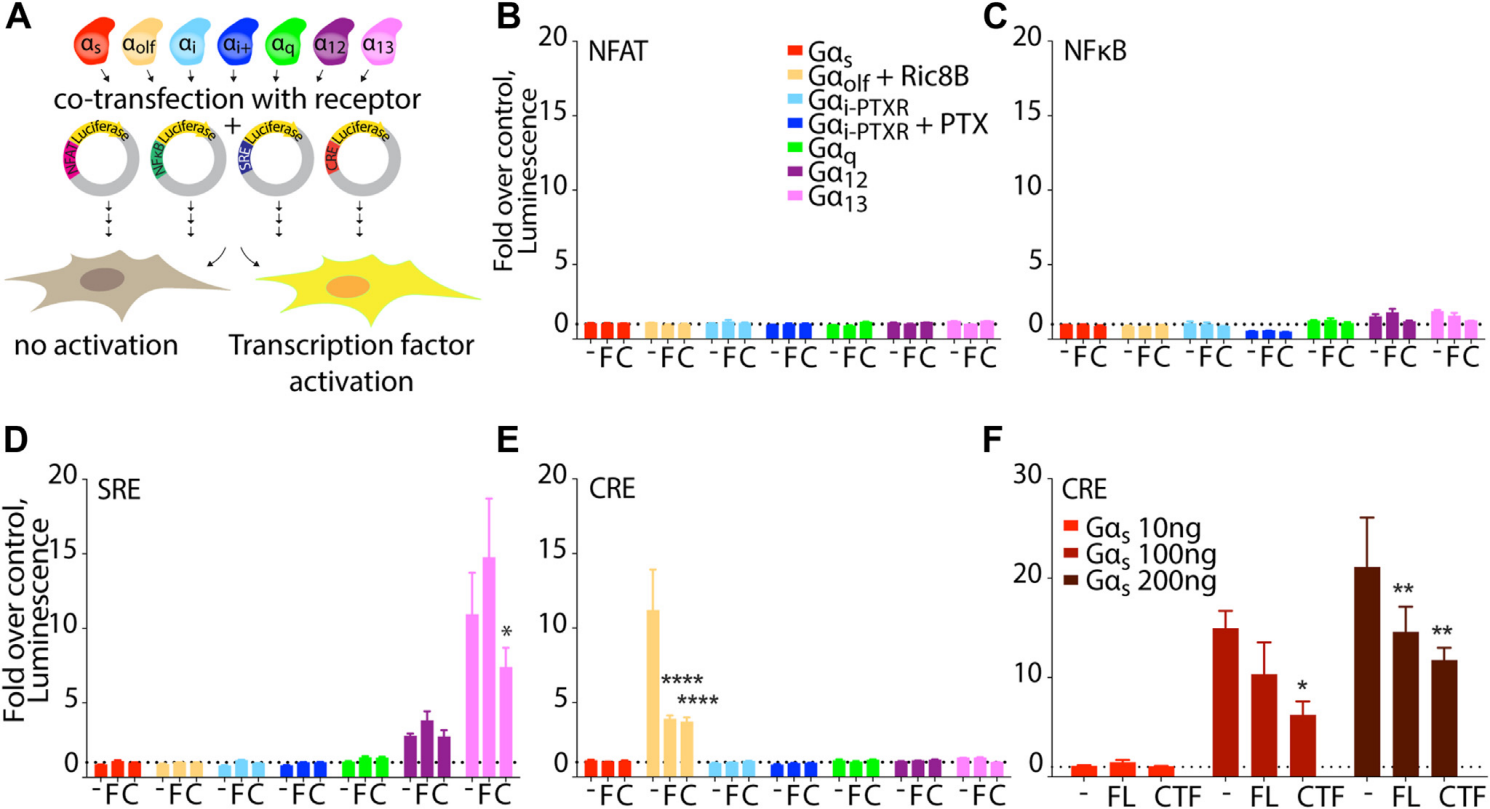
**Decreased CRE response in ADGRA3-expressing cells in a downstream readout of Gα protein screening.***A*, schematic of the Gα protein screening in HEK7GKO cells (Gα subunit-depleted, except for Gα_i/o_). One reporter gene plasmid (300 ng) containing luciferase under the control of a transcription factor response element (NFAT/NFκB/SRE/CRE) was cotransfected with ADGRA3 FL, CTF or empty vector, as well as one Gα subunit at a time (Gα_s/olf+Ric8B/i-PTXR/i-PTXR+PTX/q/12/13_) (10 ng). Downstream pathway activity was measured as an increase (or decrease in the case of Gα_i_ family proteins) in luminescent emission signal at 525 nm. *B*–*E*, screening results stratified by each transcription factor reporter gene. *F*, CRE reporter gene assay with increased amounts of Gα_s_ cDNA (10 ng, 100 ng, 200 ng) in HEK7GKO cells, cotransfected with ADGRA3FL, CTF, or empty vector. *B*–*F*, data presented as fold over no G protein control (*dashed line*) from three (*B*–*E*) or five (*F*) independent experiments in technical triplicates ± SEM. Statistics: *B*–*F*, two-way ANOVA, followed by Tukey’s correction for multiple testing. *Asterisks* denote comparisons to no G protein empty vector control: ∗*p* < 0.05, ∗∗*p* < 0.01, and ∗∗∗∗*p* < 0.0001. The full result of the statistical analysis can be found in Table S3. CTF, C-terminal fragment; CRE, cAMP response element; FL, full length; NFAT, nuclear factor of activated T cells; NFκB, nuclear factor kappa-light-chain-enhancer of activated B cells; PTX, pertussis toxin; PTXR, pertussis toxin resistant; Ric8B, RIC8 guanine nucleotide exchange factor B; SRE, serum response element.

To assess the ability of ADGRA3 to activate heterotrimeric G proteins, we first screened for its capacity to engage each of the four major G protein–dependent pathways. G-protein engagement by ADGRA3 has been suggested from previous studies based on coimmunoprecipitation (20, 21), but the actual ability of ADGRA3 to activate G protein–dependent signaling has never been described. To address each of the G-protein families separately, we used a HEK293A-derived CRISPR knock-out cell line devoid of Gα subunits, α_s/olf, z, q/11, 12/13_ (HEK7GKO) (42) expressing solely Gα subunits of the α_i/o_ family. As a readout for pathway activity, we used four distinct luciferase reporter gene assays, which have been successfully used for profiling class A GPCRs and other aGPCRs to characterize constitutive (basal, ligand-independent) signaling downstream of heterotrimeric G-protein activation (43, 44, 45). In these reporter gene assays, luciferase is placed under the control of a specific transcription factor response element promoter, and receptor activity is quantified as a change in luminescence compared to empty vector control. We used the following four transcription factor systems: Nuclear factor of activated T cells (NFAT, controlled mainly by Gα_q/11_), nuclear factor κ-light-chain-enhancer of activated B cells (NFκB, as readout mainly downstream of Gα_q/11_ and Gα_12/13_), serum response element (SRE, as readout mainly downstream of Gα_12/13_ and Gα_q/11_), and cyclic adenosine monophosphate (cAMP) response element (CRE, controlled mainly by Gα_s_ and Gα_i_. By reintroducing one Gα subunit at a time, using reporter gene assays in the HEK7GKO cells, we screened ADGRA3 FL and CTF constructs, for the G-protein selectivity. (Fig. 2*A*). As positive controls, we used receptors known to couple to the targeted Gα isoform in the HEK7GKO cells (Fig. S1) and as previously shown (42). We did not observe any significant activity changes upon transient expression of either of the two constructs in the NFAT or NFκB cotransfected with any Gα subunit and at relevant assay windows (44, 46) (Fig. 2, *B* and *C*). Compared to ADGRA3 FL, ADGRA3 CTF slightly decreased the SRE luminescence upon cotransfection with Gα_12_ and Gα_13_ (Fig. 2*D*). In the CRE reporter gene assay, both ADGRA3 FL and CTF decreased luminescence in Gα_olf_-expressing cells (with the addition of Ric-8B to chaperone Gα_olf_ expression) (Fig. 2*E*). As Gα_olf_ is a member of the Gα_s_ family of adenylyl cyclase-stimulating Gα subunits, the decrease in CRE activity suggests activation of endogenous Gα_i/o_ proteins. By titrating in a Gα_s_ subunit construct at two additional concentrations (100 ng and 200 ng, Fig. 2*F*), we confirmed that CRE activity was suppressed by both ADGRA3 FL and CTF, also when cotransfected with Gα_s_ (Fig. 2*F*). Thus, the ADGRA3-mediated counteraction of Ga_olf/s_ signaling to CRE-dependent transcription, suggests that ADGRA3 activates endogenous Gα_i_ family proteins.

### ADGRA3 signals through both G_i_ and G_s_ proteins

**Figure 3:**
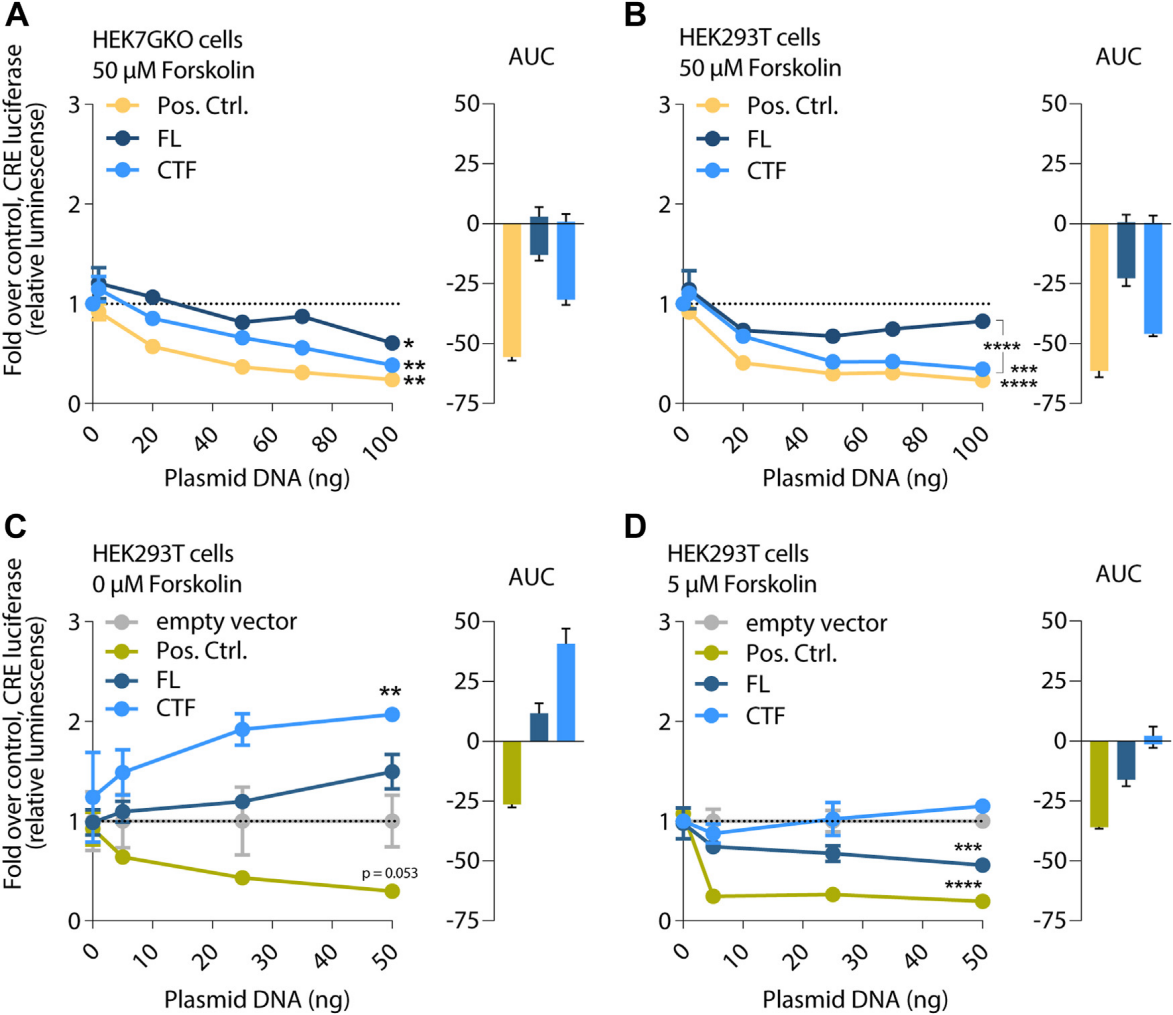
**ADGRA3 signals *via* Gα_i_ and Gα_s_ with a stronger activity of CTF relative to FL receptor.***A*–*D*, CRE reporter gene assays with increasing gene doses of ADGRA3 FL, CTF, and positive control for Gα_i_ activity; (*A*) HEK7GKO cell line or (*B*) HEK293T cells were cotransfected with Gα_sΔ10_ (*C* terminally truncated Gα_s_, which is cAMP-inducing, but unable to couple to G proteins) and pretreated with 50 μM forskolin (*high*) to stimulate adenylyl cyclase. D_2_R serves as a control for constitutive Gα_i_ activity. *C*, HEK293T cells received no adenylyl cyclase stimulation. *D*, HEK293T cells prestimulated with 5 μM forskolin (*low*). *C* and *D*, BILF1 serves as a control for constitutive Gα_i_ activity. *A*–*D*, luminescence was measured at 525 nm. Data are presented as mean fold over empty vector control ± SEM of three independent experiments of technical triplicates. *A* and *B*, *dotted line* displays 0 ng ADGRA3 cDNA point (100 ng empty vector); cDNA weight was adjusted to 100 ng/well with empty vector during transfection. *C* and *D*, empty vector was tested experimentally in parallel to ADGRA3 with increasing gene dose (0–50 ng). cDNA weight was not adjusted for each gene dose, but data were normalized to the corresponding empty vector cDNA weight. *A*–*D*, area under the curve (AUC) is visualized next to each graph ± standard error. Statistics: (on the last data point) one-way ANOVA followed by Tukey’s correction for multiple testing. *Asterisks* show comparison to empty vector control unless otherwise indicated by brackets; ∗*p* < 0.05, ∗∗*p* < 0.01, ∗∗∗*p* < 0.001, and ∗∗∗∗*p* < 0.0001. The full result of the statistical analysis can be found in Table S3. AUC, area under the curve; CRE, cAMP response element; CTF, C-terminal fragment; D_2_R, dopamine receptor D_2_; FL, full length; PTX-S1, pertussis toxin subunit 1.

Next, we further investigated the Gα_i_-mediated signaling elicited by ADGRA3 by cotransfecting the CRE-luciferase reporter gene with increasing plasmid amounts of either of the two ADGRA3 constructs (FL and CTF) into the HEK7GKO cell line. To elevate intracellular cAMP levels, the cells were preincubated with forskolin (50 μM), a direct activator of adenylyl cyclase, and transfected with C terminally truncated Gα_s_ (Gα_sΔ10_), which fully complements adenylyl cyclase activity to induce cAMP, but has abolished receptor coupling (44). The dopamine receptor D_2_ served as a positive control for Gα_i_ coupling. We found that ADGRA3 FL and CTF decreased the forskolin-induced CRE signal in a gene dose-dependent manner, supporting the concept of constitutive receptor activity toward Gα_i/o_ proteins (Fig. 3*A*). The two receptor constructs were expressed at the cell surface at comparative levels (Fig. S2, *A* and *B*). To rule out a cellular bias of the CRISPR cell line (HEK7GKO) in driving the Gα_i/o_ protein activity, we repeated the experiment in WT HEK293T cells. Again, ADGRA3 FL and, to a greater extent, CTF decreased CRE activity even when the full panel of endogenous Gα subunits was present (Fig. 3*B*). Consulting the area under the curve (AUC), the two graphs (bar plots Fig. 3, *A* and *B*) show very similar patterns between the two cell types.

We proceeded to omit the forskolin-induced prestimulation of adenylyl cyclase. In this setting, using HEK293T cells with endogenously expressed Gα_s_ and Gα_i_, ADGRA3 CTF significantly increased the CRE luminescence compared to empty vector transfections (Fig. 3*C*). Again, the CTF construct appeared more active than FL ADGRA3, only trending to increase the CRE luminescence. The constitutively activated Epstein-Barr virus-encoded receptor, BILF1, was included as a positive control for Gi activity (47, 48), and negatively impacted the CRE response in comparison to empty vector control (*p* = 0.053) (Fig. 3*C*). Collectively, this suggests Gs and Gi activity of ADGRA3 in this setting without a pre-stimulatory pressure on adenylate cyclase.

To explore the balance between the apparent divergent Gs-Gi activities, we decreased the forskolin concentration from 50 μM to 5 μM, a concentration eliciting a submaximal CRE response, in the HEK293T cell line (Fig. S2*C*). Under these conditions, with reduced adenylyl cyclase activity, ADGRA3 FL decreased the CRE luminescence comparable to that seen after the high forskolin preincubation (Fig. 3*D*), and so did the positive control, BILF. In contrast, the CRE response downstream from the CTF oscillated around zero at low forskolin level (Fig. 3*D* gene dose and area under the curve), suggesting a stronger Gs relative to Gi balance of CTF (*i.e*., in the absence of NTF) than FL receptor.

### Knocking out G**α**_s_ abolishes the positive CRE signal and increases CRE inhibition

**Figure 4:**
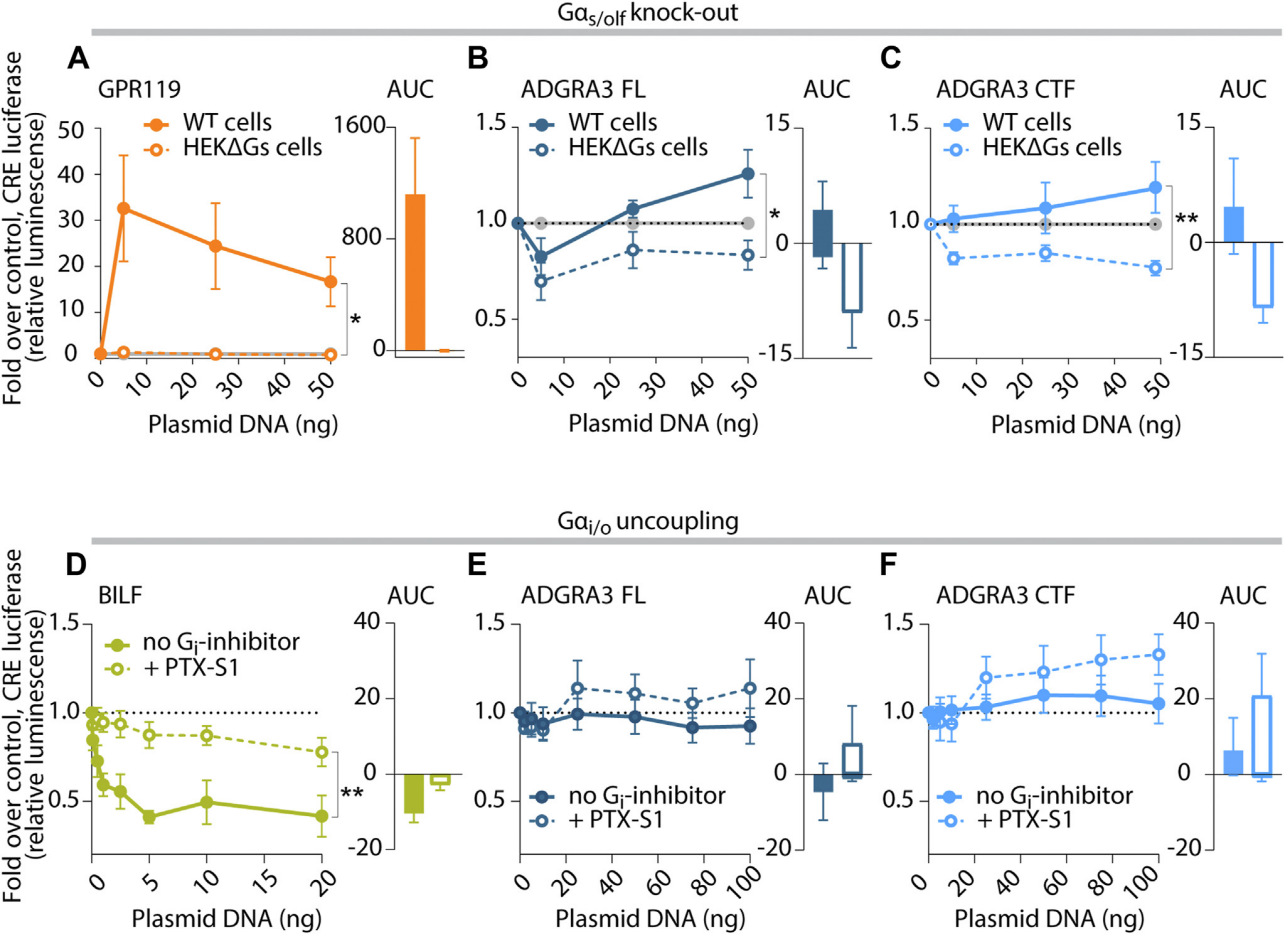
**Modulation of cellular Gα_s/olf_ or Gα_i/o_ confirms ADGRA3 signaling through both axes**. *A*–*C*, CRE reporter assays in HEK293A “parental” cells and CRISPR-engineered Gα_s/olf_ knock-down cell line, HEKΔGs. *A*, GPR119 serves at positive control for Gα_s_-coupled signaling. *B*, ADGRA3 FL and (*C*) CTF gene doses in the presence of 5 μM forskolin (*low*). *D*–*F*, CRE reporter assays in HEK293T cells with or without Gα_i_ uncoupling by PTX-S1 cotransfection and in the presence of 5 μM forskolin (*low*). *D*, BILF serves as control for constitutive Gα_i_ activity. Gene doses of (*E*) ADGRA3 FL and (*F*) CTF. *A*–*F*, Data are presented as mean fold over empty vector control ± SEM. *A*–*C*, empty vector was tested experimentally in parallel to ADGRA3 with increasing gene dose (0–50 ng), and data were normalized to the corresponding empty vector data point, n = 4. *D*–*F*, *dotted line* displays 0 ng ADGRA3 cDNA point (100 ng empty vector) and total cDNA weight was adjusted to 100 ng/well with empty vector during transfection, n = 7. *A*–*F*, area under the curve (AUC) is visualized next to each graph ± standard error. Statistics: (on last data point) (*A*–*C*) one-way ANOVA with Tukey’s *post hoc* test and (*D*–*F*) Student’s paired two-tailed *t* test on plotted data. ∗*p* < 0.05 and ∗∗*p* < 0.01. The full result of statistical analysis can be found in Table S3. AUC, area under the curve; PTX-S1, pertussis toxin subunit 1; FL, full length; CTF, C-terminal fragment; CRE, cAMP response element.

To confirm Gα_s_-mediated increase in CRE activity from ADGRA3, we used a HEK293A-derived CRISPR KO cell line devoid of the Gα_s_ family subunits (HEKΔGs) (49) and repeated the CRE reporter assay. As a positive control for Gα_s_-mediated CRE signaling, we included GPR119, a cannabinoid receptor-like class A GPCR (50, 51). In the parental cell line, expressing the full panel of Gα subunits, we saw a strong positive CRE response, even at low gene doses, from GPR119, which was completely abolished in the HEKΔGs cells (Fig. 4*A*). For ADGRA3 FL, we observed an oscillation around baseline in the parental cell line supplemented with low (5 μM) forskolin, which was downshifted in the HEKΔGs cells, reflective of increased Gα_i_ activity upon Gα_s_ depletion, albeit signaling was weak (Fig. 4*B*). For CTF, we observed an increase in the CRE signal at the same low forskolin level (Fig. 4*C*) in the parental cell line (with Gα_s_), however, in the Gα_s_-depleted cells, this increase was abolished, and the CRE luminescence further decreased, again reflecting more Gα_i_ signaling activity in the absence of Gα_s_. This suggests that ADGRA3 CTF balances toward Gs signaling in HEK293A WT cells with this level of basal CRE activity.

### Cellular differences in CRE response reveal ADGRA3 Gs activity requires a certain pre-pressure on Gs

Noticeably, we observed differences in the CRE responses, both in basal levels and, in particular, in response to forskolin between the different cell lines (Fig. S3). The HEK293A cells had a mean basal CRE response of ∼1000 relative luminescence units, which increased around 30-fold upon the addition of 5 μM forskolin. The basal CRE response was reduced to half in HEKΔGs cells (derived from HEK293A) and increased less than 3-fold with the same forskolin addition. We attribute the modest CRE response in the HEKΔGs to the hampered Gα_s_ signaling axis. However, comparing the two cell lines with WT levels of G proteins, we found that HEK293T had a much higher basal CRE activity, almost six times higher than HEK293A. Adding 5 μM forskolin increased this further by 20-fold. Our results suggest that ADGRA3 is more prone to mediate an increase in CRE response in cells with a certain prepressure on the Gs signaling axis, that is, HEK293A supplemented with 5 μM forskolin, or unstimulated HEK293T cells. If the initial CRE activity is higher than the “Gs signaling zone”, as in HEK293T cells stimulated with 5 or 50 μM forskolin, ADGRA3 switches to Gα_i_ signaling, decreasing the CRE luminescence (Fig. S3).

### Inhibition of G**α**_i_ ablates the CRE signal decrease

To control for Gα_i_ coupling, we exploited *Bordetella pertussis* toxin (PTX) that ADP-ribosylates all members of the Gα_i/o_ family (except for Gα_z,_ which lacks a cysteine residue four positions from its C terminus), thereby uncoupling them from their receptors (52). In the presence of forskolin, the decrease in CRE signaling elicited by the Gα_i_-coupling receptor, BILF1, was ablated, as shown by a “flattening” of the curve, when the active S1 subunit of PTX (PTX-S1) was coexpressed (Fig. 4*D*). Having verified that the assay worked as expected, we repeated the setup with increasing ADGRA3 FL gene dose, and again we observed a CRE response oscillation just below baseline in the cells with low forskolin, but upon PTX-S1 cotransfection the CRE signal was up-shifted (Fig. 4*E*). Similarly, increasing gene dose of ADGRA3 CTF, in the presence of low forskolin, showed a CRE activity just above baseline which increased upon Gα_i_-uncoupling by PTX-S1, supporting Gs activity in the absence of an active Gα_i_ (Fig. 4*F*). Collectively, our data suggest that ADGRA3 is capable of signaling through both the Gi and Gs axes to modulate CRE activity and that CTF shows a stronger signaling response than FL.

### Truncating the tethered agonist of ADGRA3 abolishes G protein–mediated signaling

**Figure 5:**
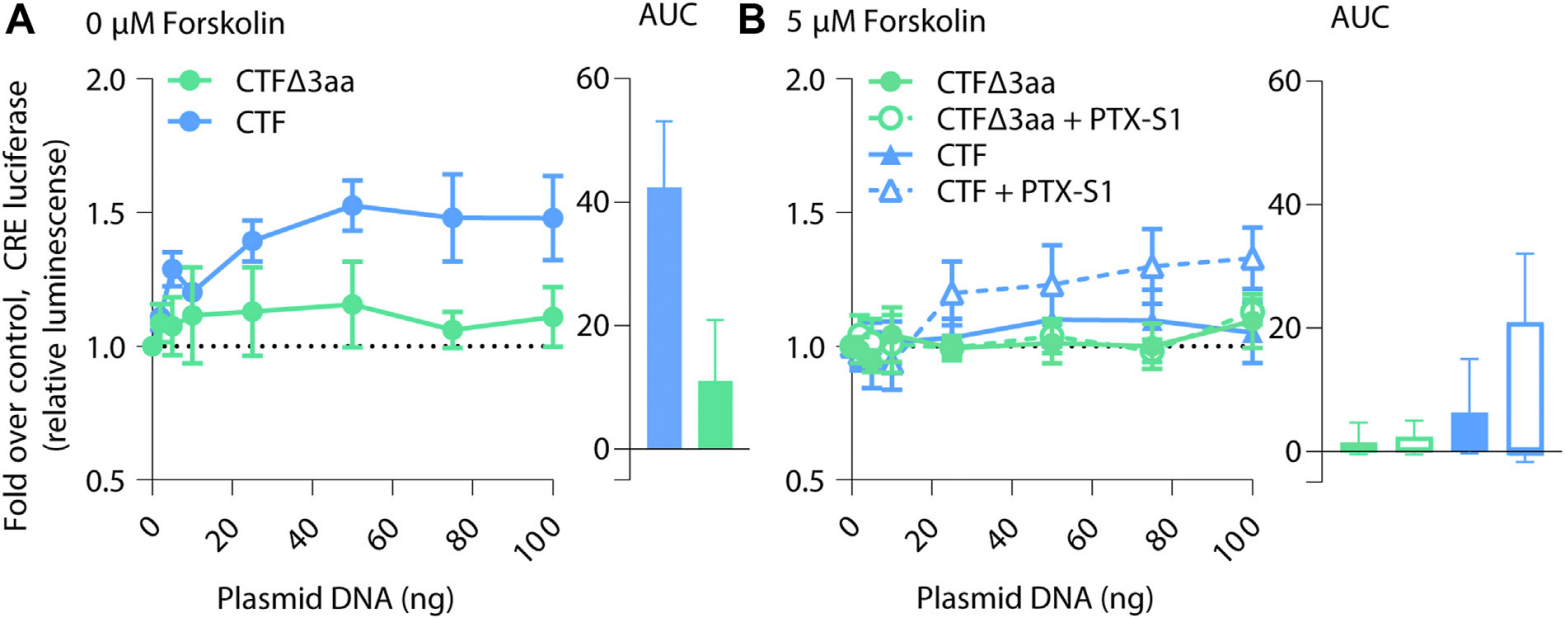
**Truncation in the ADGRA3 tethered agonist sequence abolishes G-protein activation.***A*, CRE reporter gene assay with increasing gene-doses of ADGRA3 CTF and CTFΔ3aa in HEK293T cells in the absence of forskolin. *B*, CRE luminescence from ADGRA3 CTFΔ3aa after prestimulation with 5 μM forskolin (*low*), with and without Gα_i/o_ uncoupling by PTX-S1 cotransfection. ADGRA3 CTF CRE response reprinted from Fig. 4*F* (*sky blue triangles*) for comparison. Data are presented as mean fold over empty vector control (*dotted line*) ± SEM, n ≥ 4. Area under the curve (AUC) is visualized next to each graph ± standard error. Statistics: Student’s paired two-tailed *t* test on plotted data (on last data point). The full result of statistical analysis can be found in Table S3. AUC, area under the curve; CRE, cAMP response element; CTF, C-terminal fragment; FL, full length; PTX-S1, pertussis toxin subunit 1.

For several aGPCRs, the sequence immediately downstream from the GPS cleavage site works as an intramolecular tethered agonist to activate downstream signaling (28, 29, 30, 31). Since we detected a difference in the G-protein signaling response of the artificially designed CTF compared to ADGRA3 FL, we addressed the role of the tethered agonist on ADGRA3 signaling properties. We deleted the first three amino acid residues after the GPS motif in the CTF construct (CTFΔ3aa) (Fig. 1, *A* and *B*) (29) and tested the activity using the CRE reporter gene assay. In contrast to the CTF construct with an intact tethered agonist, CTFΔ3aa did not increase the CRE luminescence with increasing receptor gene dose (Fig. 5*A*). After low forskolin preincubation (5 μM), the elevated CRE response by CTF, and following shift upward by PTX-S1 cotransfection, was not copied by the truncated CTFΔ3aa (Fig. 5*B* with data reprinted from Fig. 4*F* for comparison), suggesting that the three-amino-acid deletion abolished CRE response. This indicates that for ADGRA3 CTF, the sequence corresponding to the class-defined tethered agonist is pivotal for its G protein–mediated signaling.

### Gi-protein coupling by ADGRA3 does not depend on cellular DVL expression

**Figure 6:**
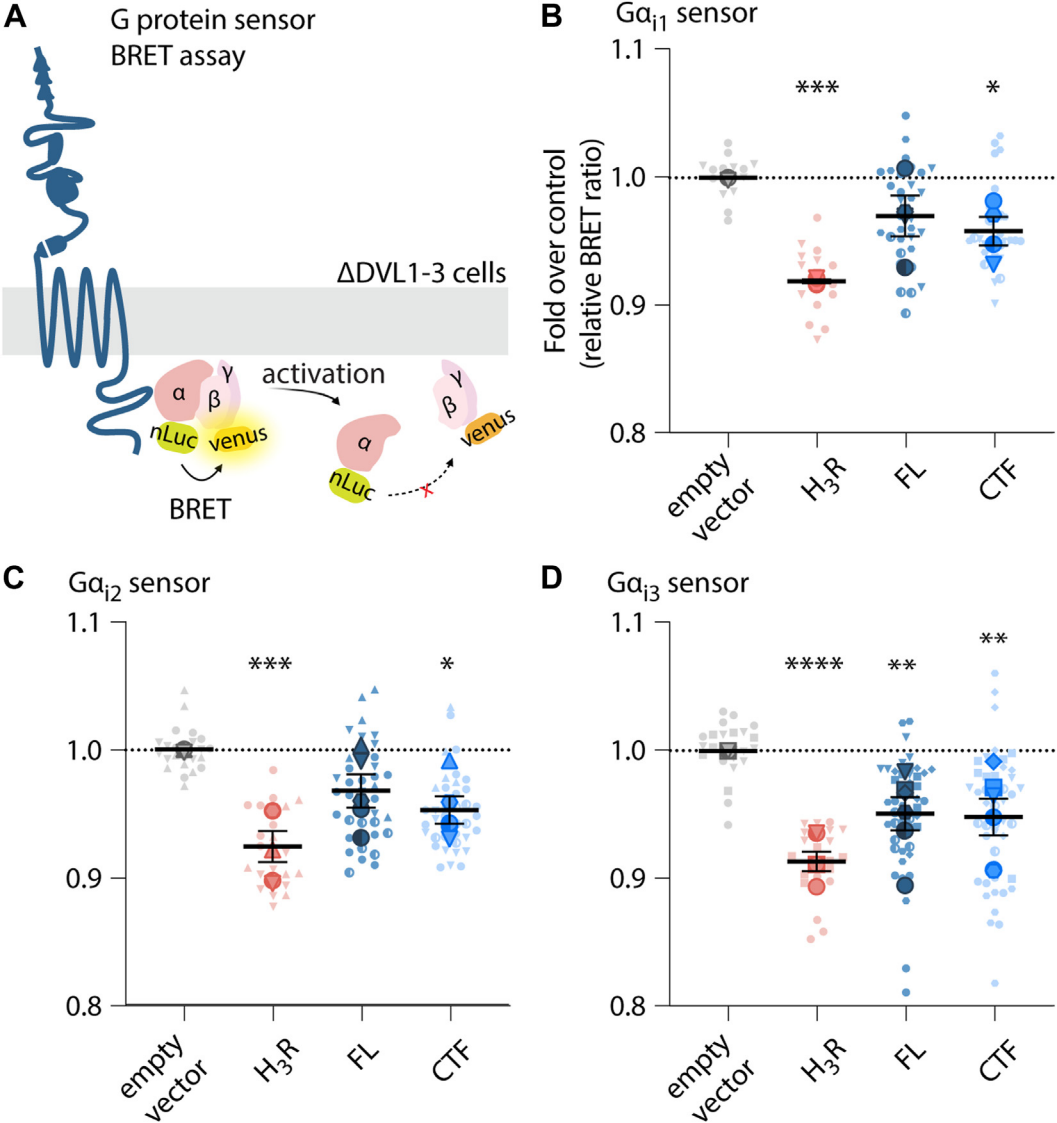
**G-protein activation by ADGRA3 CTF is sustained in the absence of DVL1-3.***A*, schematic outline of G-protein activity BRET sensor system. Upon activation, the heterotrimeric G-protein complex dissociates, and the BRET signal between nLuc-tagged Gα (donor) and cp-Venus–tagged Gγ (acceptor) ceases. The BRET sensors are cotransfected with the depicted receptors into ΔDVL1-3 cells. *B*–*D*, Gα_i1-3_ sensors cotransfected with ADGRA3 FL and CTF. H_3_R serves as positive control for constitutive Gα_i_ activity. Empty vector control is pcDNA3.1+. Data are presented as BRET ratio, emission [venus/nLuc], shown as fold over empty vector control for each transfection in “SuperPlots” (85): *Small*, *light*-*colored data points* show the distribution of technical replicates, and *large, darker data points* on *top* show the biological replicates (n ≥ 4) and are the basis of mean ± SEM overlayed and statistical testing. Different data point shapes visualize which technical and biological replicates belong to the same experiment. Statistics: one-way paired ANOVA followed by Tukey’s correction for multiple testing. *Asterisks* show the comparison to empty vector control; ∗*p* < 0.05, ∗∗*p* < 0.01, ∗∗∗*p* < 0.001, and ∗∗∗∗*p* < 0.0001. The full result of the statistical analysis can be found in Table S3. BRET, bioluminescence resonance energy transfer; CTF, C-terminal fragment; DVL, disheveled; FL, full length; H3R, histamine receptor type 3.

Having established that ADGRA3 engages both Gs and Gi signaling pathways and considering that previous studies show DVL redistribution by the receptor (11, 21), we next investigated whether the two pathways might be interrelated. To answer this question, we addressed the Gi activation by ADGRA3 in HEK293T cells depleted for all DVL isoforms (ΔDVL1-3 cells) (34) by applying a G protein activity sensor bioluminescence resonance energy transfer (BRET) system based on a tricistronic vector (G-CASE), which has been described for other constitutive active GPCRs (53, 54). Upon receptor coupling, the heterotrimeric G protein complexes dissociate, and the BRET signal between nLuc-tagged Gα and cp-Venus–tagged Gγ decreases (Fig. 6*A*). We addressed the three main Gα_i_ proteins (Gα_i1-3_) and found that ADGRA3 CTF was able to engage all Gα_i_ proteins (Gα_i1_, Gα_i2_, and Gα_i3_) as seen by a significant decrease in the BRET ratio compared to empty vector control (Fig. 6, *B*-*D*). The FL receptor, ADGRA3 FL, only elicited BRET sensor activity when coexpressed with Gα_i3_. As a positive control, we used the histamine receptor type 3 (H_3_R), based on its constitutive Gα_i_ coupling (53). Collectively, this suggests that ADGRA3 is capable of activating Gai signaling axes independently of DVL presence.

### ADGRA3 does not engage WNT/**β**-catenin signaling

**Figure 7:**
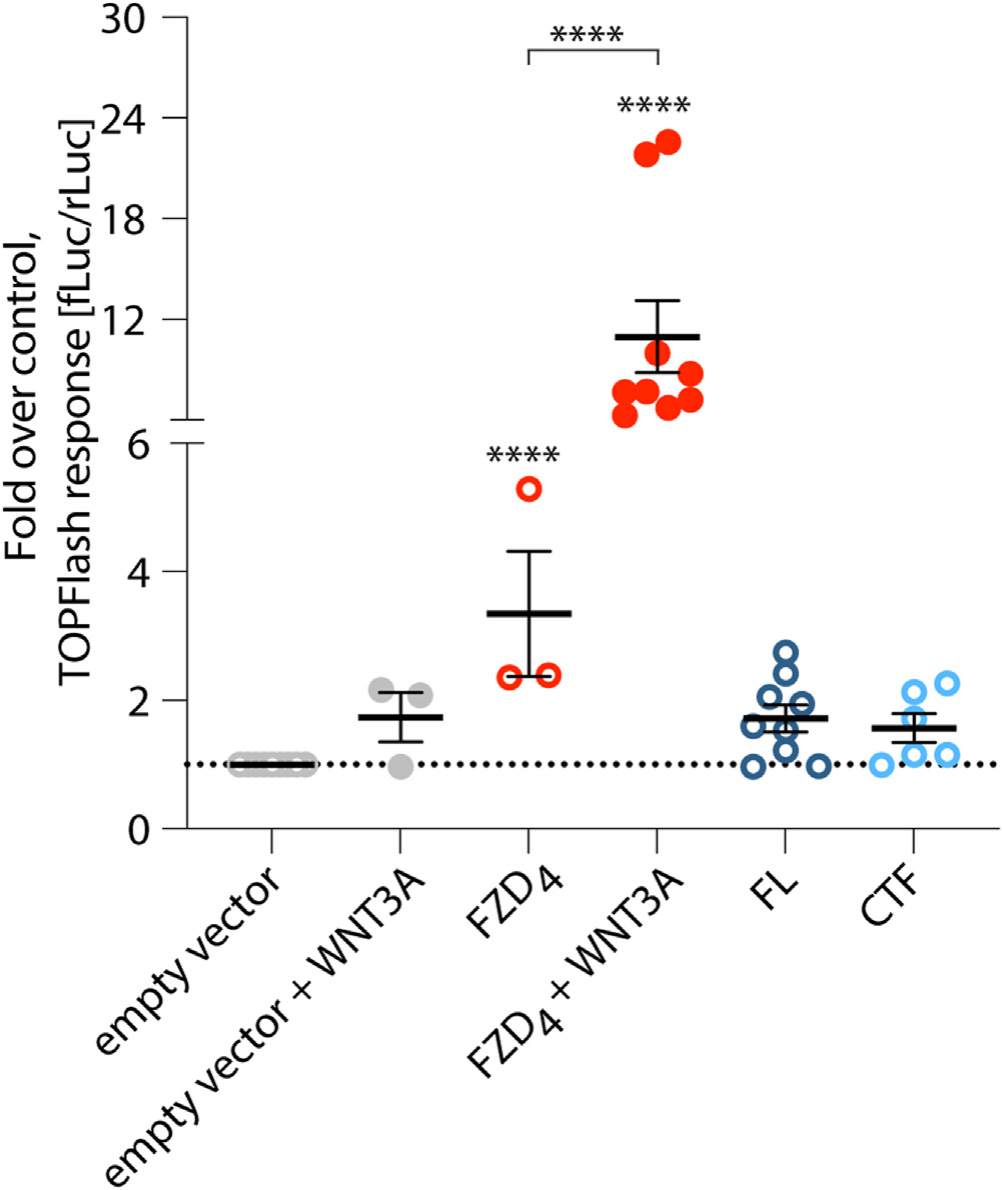
**ADGRA3 does not engage WNT/β-catenin signaling.** Quantification of the β-catenin responsive TOPFlash assay in ΔFZD_1–10_ cells, co-transfected with ADGRA3 FL, CTF or FZD_4_. FZD_4_ serves as positive control and is shown with or without 1 μg/ml WNT3A ligand stimulation. TOPFlash signal was read as [580 nm (fLuc)/480 nm (rLuc)]. One datapoint is the average of one independent transfection, n = 3–9 transfections, and data are presented as fold over empty vector control (pcDNA) with mean ± SEM indicated. Statistics: one-way ANOVA followed by Tukey’s correction for multiple testing. Asterisks denote comparisons to negative control, unless brackets indicate otherwise; ∗∗∗∗*p* < 0.0001. The full result of the statistical analysis can be found in Table S3. CTF, C-terminal fragment; FL, full length; fLuc, firefly luciferase; FZD, frizzled; rLuc, renilla luciferase.

DVL is an important early mediator of both β-catenin–dependent and β-catenin–independent WNT signaling pathways (28), and ADGRA3 was previously suggested to inhibit WNT/β-catenin signaling at the β-catenin transcriptional level in WNT3A-stimulated cells (11, 15, 21). To address the function of ADGRA3 in cells without a prevailing pressure on the WNT signaling pathways, we investigated the transcriptional activation downstream of β-catenin and TCF/LEF (T-cell factor/lymphoid enhancer factor) using a TOPFlash reporter gene assay in the absence of frizzled expression (ΔFZD_1-10_ cells) (55). Compared to the empty vector control, we were not able to detect an increase in the TOPFlash signal when cotransfecting with ADGRA3 FL or CTF (Fig. 7). As expected, FZD_4_ induced an increase in the β-catenin-regulated and TCF/LEF-dependent transcriptional activation, which was potentiated by the addition of its ligand WNT3A (Fig. 7).

## Discussion

Here, we worked with the most abundant transcript variant of ADGRA3 and systematically screened for pathway activation through distinct heterotrimeric G proteins. Our expression system included receptor gene overexpression and WT and engineered KO cell lines to study the influence of other previously reported pathways on G-protein signaling of ADGRA3.

The signal transduction from the 33 members of the aGPCR class spans both G protein–coupled and alternative pathways. Receptor-induced G-protein activation has been reported for more than half of the aGPCRs, and typically, one receptor pleiotropically engages more than one G protein (2, 28). For example, simultaneous Gα_s_ and Gα_i_ activation has been shown for ADGRL1 (latrophilin1) and ADGRV1 (GPR98), which also signal through Gα_q_, and for ADGRG6 (GPR126) (56, 57, 58, 59, 60). We find that ADGRA3 engages both Gs and Gi. The enhanced Gs activity in the presence of PTX, combined with the enhanced Gα_i_ in the absence of Gα_s_, supports this dual coupling and the delicate balance between Gs and Gi for ADGRA3. The phenomenon that inhibition of the inhibitory subunit (Gα_i_) by PTX strengthens the outcome of the signaling from Gs is not a novel phenomenon. It was previously described in, for instance, the β_2_-adrenergic receptor that coupled effectively to the same two Ga subunits and hence both activate and inhibit the adenylate cyclase *via* coupling to Gα_s_ and Gα_i_, respectively (61, 62), like observed here for ADGRA3 (Figs. 3 and 4).

Although the CRE response downstream of ADGRA3 is weak, we find that the CTF elicits a slightly higher signal than ADGRA3 FL (*e.g.*, Fig. 3*B*), in concordance with observations from other aGPCRs (27). The ability of ADGRA3 CTF to decrease the SRE response slightly in the presence of Gα_13_ suggests some level of pathway crosstalk at the gene expression level. Furthermore, we find the signaling to be cell-subtype–dependent (HEK293T *versus* HEK7GKO with HEK293A as parental cell line), thus reflected by the G-protein composition and possibly pathway modulators, endogenously and exogenously expressed in the cell lines.

We find the G-protein activation by ADGRA3 CTF to depend on the three amino acid positions after the homologous position of the aGPCR-conserved GPS cleavage site (Fig. 5, *A* and *B*). This is consistent with recent cryogenic electron microscopy structures of several aGPCRs coupled to their respective G proteins, showing the tethered agonists embedded within a typical seven transmembrane domain orthosteric site (63, 64, 65, 66). With the increased G-protein activation downstream from the CTF relative to ADGRA3 FL, one could speculate that CTF also elicits a tethered agonist–enhanced coupling. It should, however, be emphasized that it remains to be proven that ADGRA3 is cleaved at the noncanonic GPS (SL|S/G), although one study suggests so (21).

The implemented transcription factor- and BRET-based experiments allow us to detect downstream signal amplification and loss of donor-energy transfer, respectively, but do not reveal the subcellular location of the receptor–effector interactions. Previous work shows that ADGRA3 internalizes constitutively in a β-arrestin–independent but clathrin-mediated manner (67). It was also shown by microscopy that ADGRA3 recruits DVL to distinct plasma membrane subdomains in zebrafish embryos and that Dlg1 localized ADGRA3 to the basolateral membrane of canine kidney epithelial cells (11, 21). Further studies are therefore needed to decipher the potential compartmentalized signaling of ADGRA3.

ADGRA3 belongs to family III of aGPCRs, comprising ADGRA1 (GPR123) and ADGRA2 (GPR124). Whereas ADGRA2 has never been shown to couple to heterotrimeric G proteins, it has been extensively studied for its role in the assembly of a complex signalosome of WNT7A/B-FZD-DVL/Dlg-LRP5/6 (low-density lipoprotein receptor-related protein 5/6)-in the blood-brain barrier (6, 9, 55, 68, 69, 70, 71, 72). ADGRA1 is unique in having a very short N terminus and no GPCR autoproteolysis-inducing domain. It has received limited attention regarding signaling properties but was found to coexpress and localize with Gα_i1-3_ in a study of human pluripotent stem cell reprogramming (7). Thus, it resembles ADGRA3 according to previous data and our findings (*e.g.*, Fig. 3*B*) (21).

Early studies show that ADGRA3 modulates the WNT PCP pathway in zebrafish (11). Furthermore, ADGRA3 colocalizes with Dlg1 in cell polarity regulation (21) and was found to interact with the receptor-like tyrosine kinase in an affinity proteomics study (73). Receptor-like tyrosine kinase is a WNT coreceptor and mediator of polarized cell migration during development (74, 75). It is tempting to imagine an ADGRA3 signalosome with DVL and/or Dlg as scaffolding proteins based on previous interactions (21, 23), evolving around noncanonical WNT/PCP signaling pathway components, but this remains to be elucidated. Furthermore, the observation that ADGRA3 does not depend on DVL for Gi-protein signaling (Fig. 6, *B*–*D*) suggests that the two signaling pathways entailing G-protein activation and DVL recruitment are unrelated, implying the existence of a complex signalosome of dual function or that the two processes are independent (76).

Few other aGPCRs are implicated in WNT signaling cascades. For instance, ADGRE5 (CD97) interacts with β-catenin in adherence junctions of normal and transformed colorectal cells, yet the receptor does not regulate β-catenin-TCF/LEF–mediated transcriptional activity (77). ADGRG1 (GPR56), on the other hand, activates TCF transcription (78). Assay for transposase-accessible chromatin in acute myeloid leukemia samples revealed that high ADGRG1 level correlated with both WNT and Hedgehog signal partway activity and that ADGRG1 suppression led to downregulation, at the RNA level, of “WNT-related” genes, including *DVL1* (79). We could not detect any constitutive activation of TCF transcription (TOPFlash reporter) by ADGRA3 (Fig. 7).

In a biological context, ADGRA3 was initially identified as a marker of undifferentiated spermatogonial progenitors (17). Since then, it has been shown that ADGRA3 is expressed in both mammary progenitors (13), lacrimal gland progenitors (19), in male and female reproductive tract development (12, 18), in osteoclastogenesis (24), in the brain with upregulation after brain injury (10), as well as playing a role in several cancers (13, 14, 15, 16). Collectively, this suggests that ADGRA3 is an important player in orchestrating cell and tissue homeostasis in both development and regeneration. Here, we add functional and dual G protein–mediated signaling of ADGRA3 as a potential contributor to its biological effects.

## Experimental procedures

### Materials

Dulbecco’s modified eagle medium (DMEM) + high glucose (GlutaMAX), OptiMEM + GlutaMAX, Hank’s balanced salt solution + CaCl_2_ + MgCl_2_ (HBSS^++^), and readily heat-inactivated fetal bovine serum (FBS) were from Gibco. Penicillin-streptomycin and PBS + CaCl_2_ + MgCl_2_ (PBS^++^) were from the in-house vendor (“Sterilcentralen”). Dulbecco’s PBS was from Corning. FBS (non-heat-inactivated) was from Sigma Life Science. Poly-D lysine hydrobromide (PDL) and 37% formaldehyde were from Sigma-Aldrich. Enzyme-free cell dissociation solution was from MilliporeSigma. Trypsin-EDTA was from VWR. Lipofectamine2000 was from Invitrogen. SteadyLite reporter gene assay system (Firefly d-luciferin) was from PerkinElmer. HaloTag NanoBRET 618 ligand was from Promega. Coelenterazine-h and firefly d-luciferin were from NanoLight Technology. Enzyme-free cell dissociation solution and bovine serum albumin (BSA) were from MilliporeSigma. Recombinant human WNT3A were from R&D Systems/Bio-techne (#5036-WN). WNT3A and vehicle control were dissolved in filter-sterilized 0.1% BSA in PBS (WNT3A) or HBSS (vehicle) and kept on ice when handled.

### Plasmids

ADGRA3 FL, ADGRA3 CTF, and ADGRA3 CTFΔ3aa (all harboring an N-terminal Flag-tag; DYKDDDDK) were synthesized by GenScript and were based on the sequence of NM_145290.4 cloned into a pcDNA3.1+ vector background (used in Figs. 5 (CTFΔ3aa), 6, 7).

Codon-optimized constructs of ADGRA3 FL and CTF were used in Figure 2, Figure 3, Figure 4, Figure 5 (CTF) and 6 and were obtained from GenScript, carrying an N-terminal Flag-tag (DYKDDDDK) and a C-terminal 1D4-tag, as well as an artificial signal peptide (aSP; originating from influenza hemagglutinin) instead of the inherent one.

aSP: ATG AAG ACT ATT ATC GCA CTG AGC TAC ATT TTC TGC CTG GTG TTC GCT. The C-terminal 1D4-tag on these constructs was removed by Quikchange-PCR using the following primers:

Forward: GTG GAA ACA CGA GAC CAC AGT GTG AGG GCC CGT TTA AAC CCG C.

Reverse: GCG GGT TTA AAC GGG CCC TCA CAC TGT GGT CTC GTG TTT CCA C.

The G_i1_, G_i2_, and G_i3_ protein sensors (called G-CASE (53)) BILF construct (47) and Ga_sΔ10_ construct (44) were generated and authenticated as described elsewhere.

All sequences were confirmed with Eurofins Genomic’s DNA sequencing service.

### Data processing and transcript assembly

We obtained several deep sequenced RNA-seq datasets (GSE101521, GSE138734, GSE174478) from the Gene Expression Omnibus public dataset (Table S1). Only paired-end RNA-seq samples were included, and datasets generated without random primers were excluded. The resulting datasets included 132 brain tissue samples and 102 liver tissue samples. The raw data was mapped against the hg38 human genome using STAR (version 2.7.6a; https://bioweb.pasteur.fr/packages/pack@STAR@2.7.6a) (80) with default parameters. After sorting with SAMtools (version 1.9; https://www.htslib.org/) (81), the mapped reads were assembled into transcripts and quantified by StringTie (version v2.1.3b; https://ccb.jhu.edu/software/stringtie/) (82). StringTie parameters “read coverage” (-c), “transcript length” (-m), and “bases on both sides of a junction a spliced read has to cover” (-a) were set to minimal values to avoid missing transcripts and generating a bias. The parameter “fraction of the most abundant transcript at one locus” (-f) was lowered from the default (0.01) to 0. For all other StringTie parameters, default values were used. StringTie merge mode, which provided the reference annotation (-G), was used to generate a global, unified set of transcripts across RNA-seq samples. Quantification of the abundance of the input transcripts was then performed using parameters “expression estimation mode” (-e) and the beforehand generated merged “reference annotation transcripts” (-G). For the visualization, the longest ORF was identified for each transcript and translated to protein sequence with the Python package Biopython (version 1.80; https://biopython.org/) (83). The transcript with the longest protein sequence was then screened for protein domains with InterProScan (version 5.60–92.0; https://www.ebi.ac.uk/about/news/tag/interpro/) (84) against the Pfam database (-appl).

### Cell culture and transient transfection

HEK293T cells were obtained from ATCC (#CLR-3216). Additional cells lines with targeted gene-deletion *via* CRISPR-Cas9; HEK293A with targeted deletion of *GNAS*, *GNAL*, *GNAQ*, *GNA11*, *GNA12*, *GNA13*, and *GNAZ* (G protein knockouts, HEK7GKO); HEK293A with targeted deletion of *GNAS*, *GNAL* (HEKΔGS); HEK293T with targeted deletion of *FZ1*, *FZ2*, *FZ3*, *FZ4*, *FZ5*, *FZ6*, *FZ7*, *FZ8*, *FZ9*, *FZ10* (FZD knockouts, ΔFZD_1-10_); and HEK293T with targeted deletion of *DVL1*, *DVL2*, and *DVL3* (DVL knockouts, ΔDVL1-3), were derived, authenticated, and propagated as described elsewhere (34, 42, 49, 55).

All cell lines were propagated in cell culture flasks in a growth medium containing DMEM high glucose medium supplemented with 10% (FBS) and 1% penicillin-streptomycin and were maintained at 37 °C in a 5% CO_2_ humidified incubator. FBS was heat-inactivated at 56 °C for 30 min if not readily heat-inactivated by the manufacturer. During seeding or passage, the cells were detached using trypsin-EDTA. The absence of *mycoplasma* contamination was routinely checked using the MycoAlert *Mycoplasma* Detection Kit (BioNordika). The cells were transiently transfected using Lipofectamine2000 in Opti-MEM reduced serum medium according to the manufacturer’s recommendations. For transfection of cells in suspension, the cells were seeded onto white bottom/white wall microtiter plates, precoated with 20 μg/ml PDL for 20 min, and washed twice with PBS before cell seeding unless otherwise stated.

### G**α** subunit screen and transcription factor reporter gene assays

#### Gα subunit screen

HEK7GKO cells were plated in 12-well culture plates at a density of 3 to 4 × 10^5^ cells/well and incubated overnight. Twenty four hours after seeding, cells were transiently transfected using Lipofectamine2000 (2.3 μl/1 μg plasmid DNA) and a total of 800 ng plasmid DNA as follows: 300 ng of reporter (CRE-Luc/SRE-Luc/NFκB-Luc/NFAT-Luc), 300 ng of receptor, 1 to 200 ng of Gα subunit (Gα_s_, 10 ng (for the Gα_s_ titration 10 ng, 100 ng or 200 ng Gα_s_ was used); Gα_olf_, 100 ng; Gα_i_, 100 ng; Gα_q_, 200 ng; Gα_12_, 1 ng; Gα_13_, 10 ng; and PTX, 100 ng), and pcDNA5/FRT plasmid DNA to balance the total of DNA amount accordingly.

Twenty four hours posttransfection, the cells were washed with Dulbecco’s PBS and detached in an enzyme-free solution. Cells were centrifuged for 3 min at 500 rcf, and the cell pellet was resuspended in 200 μl assay buffer (1 × HBSS, 20 mM Hepes, 0.1% wt/vol BSA, pH 7.5). Cells were distributed into a 96-well black/white IsoPlate (PerkinElmer Life Sciences) in triplicates at a volume of 60 μl/well. Finally, 30 μl of d-Luciferin dissolved in assay buffer was added to each well at a final concentration of 2 mM. After 30 min incubation, emission was read at 525 nm, using a PHERAstar FS microplate reader (BMG LABTECH). For assays using the SRE-Luc reporter, the medium was exchanged to serum-free DMEM 6 h after transfection.

#### 50 μM forskolin-stimulated CRE reporter gene assays

HEK7GKO or HEK293T cells were seeded and transiently transfected as described for the Gα subunit screening, with 600 ng of CRE-Luc reporter, increasing amounts of the indicated receptor (10–600 ng), 160 ng Gα_sΔ10_ (for HEK7GKO cells only), and pcDNA5/FRT plasmid DNA to balance the DNA amount up to 1200 ng (1360 ng for the HEK7GKO cells). Twenty four hours posttransfection, the cells were detached and distributed into 96-well black/white IsoPlates, as described, before being stimulated with 10 μl forskolin per well to a final concentration of 50 μM for a total of 5 h. Thirty minutes before reading, d-Luciferin was added, and the plate was read as described above.

To ease the comparison of data between protocols, the receptor DNA (ng) in these experiments was scaled on the graphs to match the receptor plasmid DNA/cell ratio on the day of transfection. Data was presented as fold over the 0 ng receptor (600 ng empty vector) DNA point.

#### 0 μM and 5 μM forskolin-stimulated CRE reporter gene assays

HEK293T, HEKΔGs, or HEK293A (“parental”) cells were seeded at a density of 35,000 cells/well in PDL-coated, white 96-well microtiter plates and incubated overnight. Clear plates were prepared in parallel for ELISA. After 20 to 24 h, the media was aspirated, and the cells were transiently transfected directly in the wells using Lipofectamine2000 (0.6 μl/well) as follows (per well): 30 ng CRE-Luc reporter, cotransfected with increasing amounts of the indicated receptor or pcDNA3.1+ control (0–50 ng) in a total volume of 100 μl Opti-MEM. The total DNA amount was not balanced between the conditions. After 5 h, the transfection reaction was stopped with 100 μl/well growth media, and the cells were incubated overnight. Five hours before assay reading, the cells were supplemented with 5 μl/well forskolin or dimethylsulfoxid (vehicle) to a final concentration of 0 μM or 5 μM. Cells were washed with PBS^++^ and incubated with 100 μl of 1:1 dilution of SteadyLite:PBS^++^ for 30 min before emission was read at 400 to 700 nm using EnVision Multilabel microplate reader (PerkinElmer). Data were presented as fold over empty vector control curve (pcDNA3.1+ 0–50 ng DNA with or without 5 μM forskolin addition as indicated on the figure) for each data point.

#### PTX-cotransfected CRE reporter gene assays

Using the same transient transfection and assay protocol as above, HEK293T cells were transfected with (per well) 30 ng CRE-Luc reporter, 10 ng PTX-S1, 0 to 100 ng receptor, and pcDNA3.1+ to balance the DNA amount up to 140 ng. Data was presented as fold over the 0 ng receptor (100 ng empty vector) DNA point.

#### BRET assay *(G-CASE, G-protein sensors)*

ΔDVL1-3 cells were transiently transfected in suspension as described above, using 500 ng tricistronic Gα_i1/2/3_ sensor and 500 ng of the indicated receptor or pcDNA3.1+ control, per 1 ml cell suspension. After 48 h incubation at growth conditions, the cells were washed with HBSS, and 100 μl/well of 10 μM Coelenterazine-H was added. After 10 min incubation, the nLuc (donor) emission was read at 480/30 nm, whereas cp-Venus (acceptor) emission was read at 535/30 nm, using EnVision Multilabel microplate reader (PerkinElmer). Six reads were recorded, and the mean of the three less varying serial reads was used to calculate the BRET ratio as emission [acceptor/donor].

### Enzyme-linked immunosorbent assay

Transfected cells (see above) in clear 96-well microtiter plates were fixed in 100 μl, 3.7% formaldehyde in PBS++ for 10 min and washed twice in PBS^++^. Cells were blocked in 2% BSA (w/v) in PBS^++^ for 30 min before being incubated with an anti-FlagM1 antibody (Sigma #F3040) diluted 1:2000 in 1% BSA (w/v) in PBS^++^ for 1 h. The cells were washed three times with PBS^++^ before incubation with goat anti-mouse-HRPconjugate IgG secondary antibody (Invitrogen #31430) diluted 1:1000 in 1% BSA (w/v) in PBS^++^ for 1 h. Next, the cells were washed three times with PBS^++^ and incubated with 75 μl TMB PLUS2 (Kementec #4395A) for 0.5 to 3 min until the reaction was stopped with 0.2 M H_2_SO_4_. Absorbance was read at 450 nm using a FlexStation3 (Molecular Devices) microplate reader.

### TCF/LEF luciferase reporter assay (TOPFlash)

Each ml cell suspension of ΔFZD_1-10_ HEK293T cells was transiently transfected with 500 ng pcDNA or receptor along with 100 ng pRL-TK_Luc_ (expression control) and 400 ng of M50 Super 8x TOPFlash (Addgene #12456). Cells grown in PDL-coated, white 96-well plates were washed with 120 ml HBSS/well 24 h posttransfection and incubated with FBS-free DMEM supplemented with 10 nM of the porcupine inhibitor C59 (Abcam# Ab142216). Four hours later, 1 μg/ml WNT3A or vehicle was added to the cells. Forty eight hours posttransfection, cells were washed with HBSS and lysed in 30 μl of Promega’s dual luciferase passive lysis buffer (15 min at room temperature). Next, 20 μl luciferase assay reagent was added to each well, and reporter gene activity-dependent firefly luciferase intensity was measured using a CLARIOstar (580/80 nm; 1 s integration time) or Tecan Spark (585/70 nm; 2 s) microplate reader. Next, 20 μl Stop&Glo Reagent was added to quantify Renilla luciferase emission intensity (CLARIOstar: 480/80 nm; 1 s integration time; Tecan Spark: 487/85 nm; 2 s) to control for variations in cell number and transfection efficiency. Data were presented as firefly luciferase/Renilla luciferase intensity.

### Statistical analysis

All data were analyzed using GraphPad Prism 9 (https://www.graphpad.com/). BRET ratio was calculated as emission [acceptor/donor]. One-way or two-way ANOVA using Tukey’s *post hoc* test, or student’s two-way, paired *t* test, was performed, as indicated in the Figure legends. Raw data was used for statistical analysis unless otherwise stated in the Figure legends. For all statistical tests, *p* < 0.05 was considered significant.

## Data availability

All the data needed to evaluate the conclusions in the article are present in the paper or the Supplementary Materials.

## Supporting information

This article contains supporting information.

## Conflict of interest

The authors declare that they have no conflicts of interest with the contents of this article.
