# Supplementary material for: Complex G-protein signaling of the adhesion GPCR, ADGRA3
Source: J Biol Chem. 2025 Mar 22;301(5):108441. doi: 10.1016/j.jbc.2025.108441 (PMC12059339; doi:10.1016/j.jbc.2025.108441)
Supplement: Supporting information [file mmc1.zip › supporting figures.pdf]

Supporting information

Complex G-protein signaling of the adhesion GPCR, ADGRA3

Sofie M. Bagger, Hannes Schihada, Anna L. S. Walser, Anna K. Drzazga, Lukas Grätz, Tiago Palmisano, Christina K. Kuhn, Maša Mavri, Ann-Sophie Mølleskov-Jensen, Gregory G. Tall, Torsten Schöneberg, Signe J. Mathiasen, Jonathan A. Javitch, Gunnar Schulte, Katja Spiess, Mette M. Rosenkilde.

Material included:

Supplemental Figures: S1 (page S-1), S2 (page S-2) and S3 (page S-3)

Supplemental Tables:

Supplemental Table S1 is submitted as excel-file.

Supplemental Table S2 is submitted as excel-file.

Supplemental Table S3 is submitted as a multi-sheet excel-file (legend on first sheet)

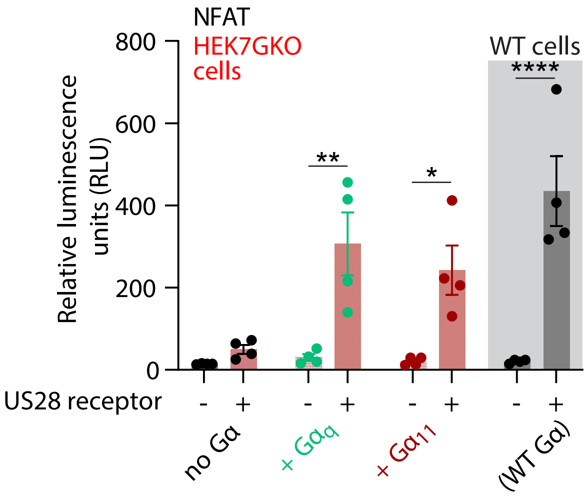

Figure S1, NFAT gene reporter assay in HEK7GKO cells without or with $G\alpha_q$ or $G\alpha_{11}$ co-transfection, shows that the US28 receptor signals via NFAT only when the targeted Gα subunits are reintroduced. NFAT reporter assay with US28 receptor in HEK293A "parental" cells with the full panel of Gα subunits included as control. Data presented as mean luminescent count ± SEM of 4 individual experiments. Statistics: One-way ANOVA with Tukey's post-hoc test. Full result of statistical analysis can be found in Table S3. *P < 0.05, **P < 0.01, and ****P < 0.0001

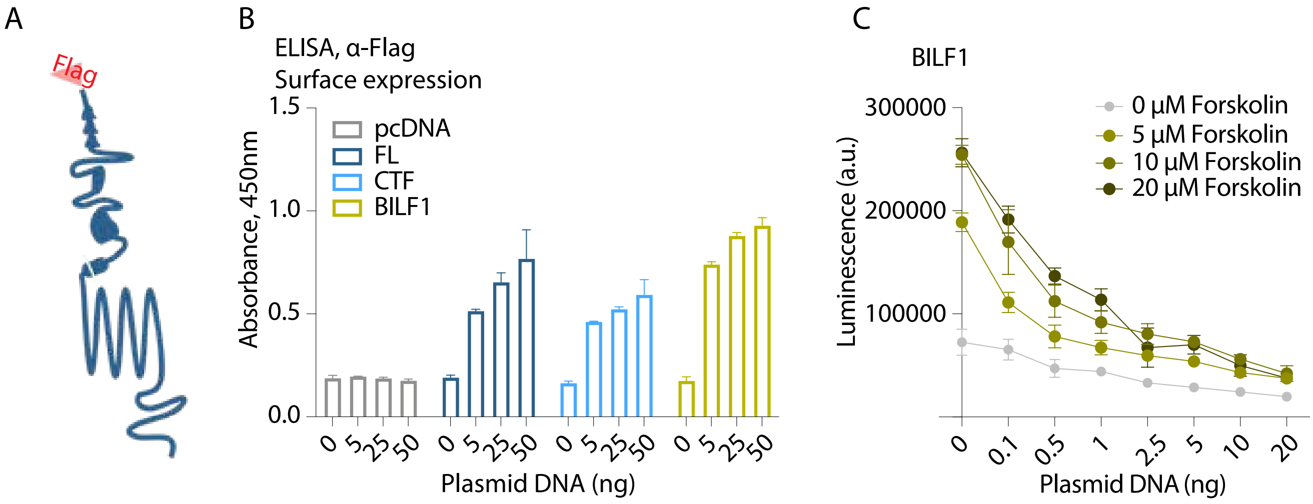

Figure S2, Cell surface expression of flag-tagged receptors and CRE reporter response following stimulation with different forskolin concentrations. (A) Schematic of N-terminally Flag-tagged ADGRA3 used for experiments in main (Figs. 2B-F and 3A-E) and (B). (B) Enzyme-linked immunosorbent assay (ELISA) against Flag-tag showing cell surface expression in unpermeabilised HEK293T cells, from same cell transfection as Figs. 3 C-D; data from one experiment with technical triplicates shown. (C) CRE reporter assays with increasing gene dose of BILF1 receptor (positive control for $G\alpha_i$ activity). Adenylate cyclase is stimulated with vehicle (0 μM), 5 μM, 10 μM or 20 μM forskolin. Data presented as mean luminescent count of 3 individual experiments ± SEM

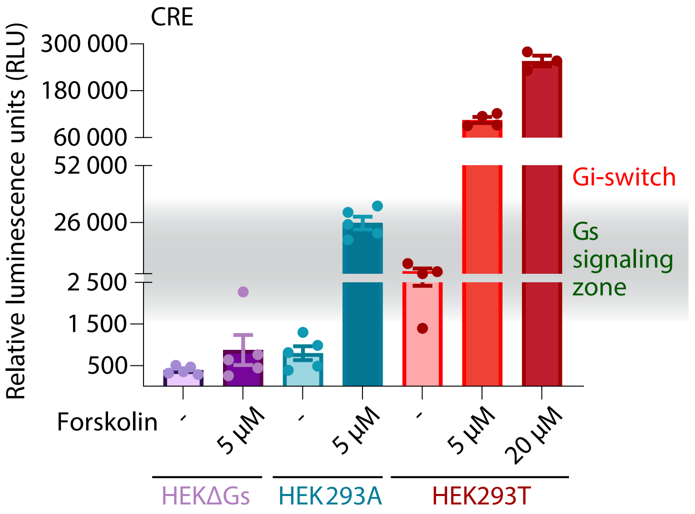

Figure S3, CRE gene reporter baseline signal response to forskolin addition is different in various HEK293-derived cell lines. No receptor is co-transfected into the cells. Data presented as mean luminescent count ± SEM of n≥3 individual experiments. Baseline data reprinted from Figs. 3, 4 and S2
